# Sports YouTube engagement as a moderator for social support on wellbeing: a mediation model with exercise behavior

**DOI:** 10.3389/fpsyg.2026.1738192

**Published:** 2026-02-13

**Authors:** Youngtaek Oh, Jun-Phil Uhm, Woong Kwon

**Affiliations:** 1 Department of Sport Science, Jeju National University, Jeju-si, Republic of Korea; 2 Department of Kinesiology, Inha University, Incheon, Republic of Korea; 3 Department of Physical Education, Jeju National University, Jeju-si, Republic of Korea

**Keywords:** exercise behavior, social support, sports engagement, wellbeing, YouTube

## Abstract

**Introduction:**

Despite the widespread belief that social support and online video-sharing social media platforms are strongly associated with exercise behavior and wellbeing, addressing this intersection has received scant attention. The purpose of this study was to examine the moderating effect of engagement with sports-related YouTube content on the relationship between social support, exercise behavior, and wellbeing.

**Method:**

The participants consisted of 226 adults (Male: 160, Female: 66) enrolled in a Sport for All education program at universities in Jeju and Gyeonggi Province, South Korea. Data were collected through an online survey administered at the recruitment sites in August 2022. The collected data were analyzed using the SPSS PROCESS Macro (Model 8) to validate the moderated mediation effect.

**Results:**

The findings indicated that social support significantly and positively associated with both exercise behavior and wellbeing, while exercise behavior was positively associated with wellbeing. Notably, the interaction between social support and sports YouTube engagement was significantly associated with exercise behavior at high and moderate levels of engagement. Furthermore, higher levels of YouTube engagement were associated with a stronger positive association between social support and wellbeing.

**Discussion:**

The results highlight the potential of digital engagement, particularly accessible sports YouTube content, to enhance exercise behavior and wellbeing in everyday life. These findings underscore the importance of leveraging social media platforms to promote public health, given the high accessibility of sports YouTube engagement. This study contributes to the interdisciplinary integration of sports media and sports psychology, providing a foundation for future digital health interventions.

## 1 Introduction

Can engagement with sports-related YouTube content serve as a proxy for a healthy lifestyle? Although excessive YouTube usage is generally perceived negatively by the public (Sokolova and Perez, 2021), sports-oriented content may have a beneficial impact by promoting physical activity and supporting the development of exercise skills through accessible at-home workout resources (Oh, 2025). As YouTube continues to evolve into a globally accessible platform for sports and fitness, such engagement may reflect individuals' commitments to a physically active lifestyle. Nevertheless, leveraging behavioral patterns in sports-related YouTube engagement as an indicator of healthy living remains largely unexplored in academic research.

A review of prior research indicates that the pursuit of a healthy and wellbeing-oriented lifestyle is a universal human aspiration (Hanawi et al., 2020). Wellbeing encompasses an individual's overall state of health, happiness, and life satisfaction, integrating physical, emotional, social, and mental dimensions (Helliwell and Putnam, 2004). Empirical studies have demonstrated that both social support (Nguyen et al., 2016; Oh, 2021) and exercise behavior (Brand et al., 2020) are positively associated with enhanced wellbeing. These findings support the existence of a strong relationship between social support and wellbeing (Kong et al., 2013), as well as between exercise behavior and wellbeing (Lesser and Nienhuis, 2020). However, it remains unclear whether these relationships can be extended to the context of sports-related YouTube engagement.

In this regard, the increasing personalization and interactivity of sports-related YouTube content suggest that digital engagement with content creators may play a significant role in shaping health-related behaviors. The visual modeling and informational support provided by these platforms can reinforce perceived social support and enhance motivation for physical activity, thereby influencing overall wellbeing. Thus, integrating these digital dynamics into the current model offers a more comprehensive framework for understanding the role of digital media in sports psychology.

Grounded in Social Cognitive Theory (Bandura, 2001) and Imitation Theory (Carpenter et al., 1998), this study aims to examine the potential influence of sports YouTube engagement on the interplay among viewers' social cognitive, behavioral, and psychological responses. Specifically, we explore how engagement with sports-related YouTube content may enhance exercise behavior and wellbeing—either through the imitation of sporting techniques or the fulfillment of personal motivations via physical activity. By investigating the moderated mediation effect of sports YouTube engagement on the relationships among social support, exercise behavior, and wellbeing, our research seeks to address a critical theoretical gap. The findings are expected to advance the field of sports social science by offering empirical insights into the intersection of digital media engagement and psychological outcomes in sports contexts.

## 2 Literature review and hypothesis development

### 2.1 Relationship between social support, exercise behavior, and wellbeing

Social support plays a critical role in enhancing psychological functioning by providing emotional, physical, and cognitive benefits through interactions with family, friends, and members of one's broader social network, including the workplace (Gülaçti, 2010). Conceptually, social support encompasses both the physical and psychological assistance provided in response to specific life circumstances, often framed within the context of empowerment. In certain social groups, it also involves the fulfillment of fundamental psychological needs such as love, loyalty, integrity, self-esteem, and a sense of belonging (Aksüllü, 2004; Tan and Karabulutlu, 2005). Given the well-established association between social support and individual wellbeing (Liu et al., 2016; Rains and Keating, 2011), the first hypothesis of this study posits a positive relationship between perceived social support and wellbeing.

Wellbeing is broadly defined as a state characterized not only by the absence of negative psychological conditions—such as depression, anxiety, anger, and fear—but also by the presence of positive emotions, physical health, a supportive environment, and the capacity for self-actualization (Adler et al., 2017). It extends beyond the mere absence of illness, encompassing subjective happiness and life satisfaction (Seligman, 2011; Seligman and Csikszentmihalyi, 2000). While wellbeing reflects the optimal functioning of positive emotional states, sustaining it does not necessarily require constant positivity. The ability to effectively regulate and cope with adverse emotions—such as disappointment, failure, and sadness—is also considered a critical component of enduring wellbeing (Oh, 2021). Prior research has consistently emphasized the significance of wellbeing, demonstrating that physical activity (Edwards, 2006), healthy lifestyle practices (Boccardi and Boccardi, 2019), and prosocial behavior (Oh, 2023) contribute positively to its development and maintenance.

Social support has been widely examined within the contexts of exercise behavior and Social Cognitive Theory. Courneya et al. (2000) emphasized that individuals' engagement in physical activity can be influenced by the active participation of close acquaintances, such as friends and colleagues. This underscores the facilitative role of social networks in encouraging exercise behavior through shared participation and mutual reinforcement. Booth et al. (2000) further suggested that exercising with familiar others may enhance motivation and adherence to physical activity routines. Empirical evidence supports the notion that social support significantly contributes to the initiation and maintenance of exercise behavior (Darlow and Xu, 2011; Resnick et al., 2002). Drawing upon these findings, the present study formulated the following hypotheses:

Hypothesis 1: Social support will be positively associated with wellbeing.

Hypothesis 2: Social support will be positively associated with exercise behavior.

### 2.2 Relationship between exercise behavior and wellbeing

Exercise behavior refers to a broad spectrum of physical activities in which individuals participate, including structured exercise, organized sports, and routine daily movements. As noted by McFadden and Li (2019), the characteristics of physical activity—such as type, frequency, intensity, duration, and underlying purpose—are shaped by individual lifestyle patterns, health conditions, and environmental contexts. Conceptually, exercise behavior is recognized as a multifaceted construct, encompassing social, ecological, psychological, and genetic determinants. A substantial body of empirical research has demonstrated that individuals who engage in regular physical activity, regardless of age or background, tend to exhibit enhanced cardiorespiratory fitness, improved wellbeing, and better overall health outcomes (Fletcher et al., 2018; Oliver et al., 2020).

Regular engagement in exercise and physical activity plays a pivotal role in sustaining a healthy lifestyle, as consistently demonstrated in extant literature (e.g., Forrest et al., 2016; Warburton and Bredin, 2017). Exercise behavior has been shown to correlate positively with various psychosocial outcomes, including personal commitment (Wilson et al., 2004), school adaptation (Park et al., 2019), and psychological wellbeing (Ersöz, 2017). Building on this evidence, the present study examines the relationship between leisure-time exercise behavior and wellbeing. Accordingly, Hypothesis 3 was formulated to investigate the association between participants' exercise behavior and their wellbeing over a 1 week period.

Hypothesis 3: Exercise behavior will be positively associated with wellbeing.

### 2.3 Sports YouTube engagement and social cognitive theory, imitation theory

Over the past few decades, the proliferation of social networking platforms has markedly transformed the way individuals communicate and engage with information (Sokolova and Perez, 2021). Social media has emerged as a powerful medium for self-expression, opinion sharing, and shaping the attitudes and behaviors of younger generations. Among these platforms, YouTube has garnered particular attention for its role in facilitating social media activity and content dissemination (Sokolova and Perez, 2021). Empirical evidence suggests that social networking applications can promote health-related behaviors by enabling users to explore personal interests and present curated aspects of their identity (Vaterlaus et al., 2015). Notably, Tiggemann and Zaccardo (2015) found that exposure to visual content—such as photographs and videos encountered while searching for health-related topics—can serve as a motivational catalyst, encouraging users to adopt healthier lifestyle practices.

YouTube-based sports programs offer a multifaceted array of resources, including instructional coaching videos, standardized exercise protocols, motivational content, and demonstrative coaching and professional expertise from fitness influencers that provide structured exercise guidance. These digital materials not only provide accessible guidance on physical activity but also function as potent motivational cues for individuals aiming to initiate or sustain health-promoting exercise behaviors (Ramchandani et al., 2014). The impact of such content is particularly pronounced among younger cohorts, who demonstrate a heightened propensity to engage with social media platforms as sources of health-related information and behavioral modeling (Sokolova and Perez, 2021). A growing body of empirical research has substantiated the efficacy of YouTube sport-related content in promoting home-based physical activity. For instance, McDonough et al. (2022) reported that exposure to fitness-oriented YouTube videos significantly enhanced users' motivation to participate in high-intensity interval training and resistance exercises. Similarly, Sokolova and Perez, 2021 found that active interaction with YouTube fitness channels contributed to increased exercise motivation among regular fitness participants.

The present study seeks to elucidate the relationship between engagement with sports-related YouTube content, individuals' cognitive understanding of the importance of exercise behavior, and their intention to emulate the skills while reinforcing the cognitive appraisal of social support through digital interaction. This investigation is grounded in the theoretical frameworks of Social Cognitive Theory and Imitation Theory. According to SCT, behavioral motivation is acquired through observational learning, wherein individuals internalize modeled actions that are perceived to yield desirable outcomes (Bandura, 2001). When observers deem the behavior portrayed by a model as effective or beneficial, the associated information is cognitively encoded for future recall and behavioral reproduction. This observational process is influenced by multiple motivational factors, including sustained interest, cost–benefit appraisal, social comparison, self-efficacy assessment, and alignment with personal standards (Sokolova and Perez, 2021). Within this framework, Anderson et al. (2006) demonstrated that social support significantly contributes to self-efficacy and self-regulatory capacity, thereby exerting a meaningful influence on physical activity engagement. These theoretical insights provide a robust foundation for examining the role of social support in shaping exercise-related outcomes in the current study.

Research on human imitation represents one of the most conceptually rich and interdisciplinary domains within cognitive and social sciences, offering critical insights into the mechanisms underlying human learning, communication, and social interaction (Garrels, 2005). Although imitation has traditionally been perceived as a rudimentary or juvenile behavior, contemporary scholarship has reframed it as a foundational cognitive capacity intricately linked to language acquisition, cultural transmission, and theory of mind across diverse academic disciplines (Hurley and Chater, 2005). Imitative behavior encompasses both visual mimicry and idealized representations of shared actions and gestures, and is closely associated with internal mental states—such as desires, beliefs, intentions, and goals—that facilitate the prediction and interpretation of human behavior (Hurley and Chater, 2005).

**Figure 1:**
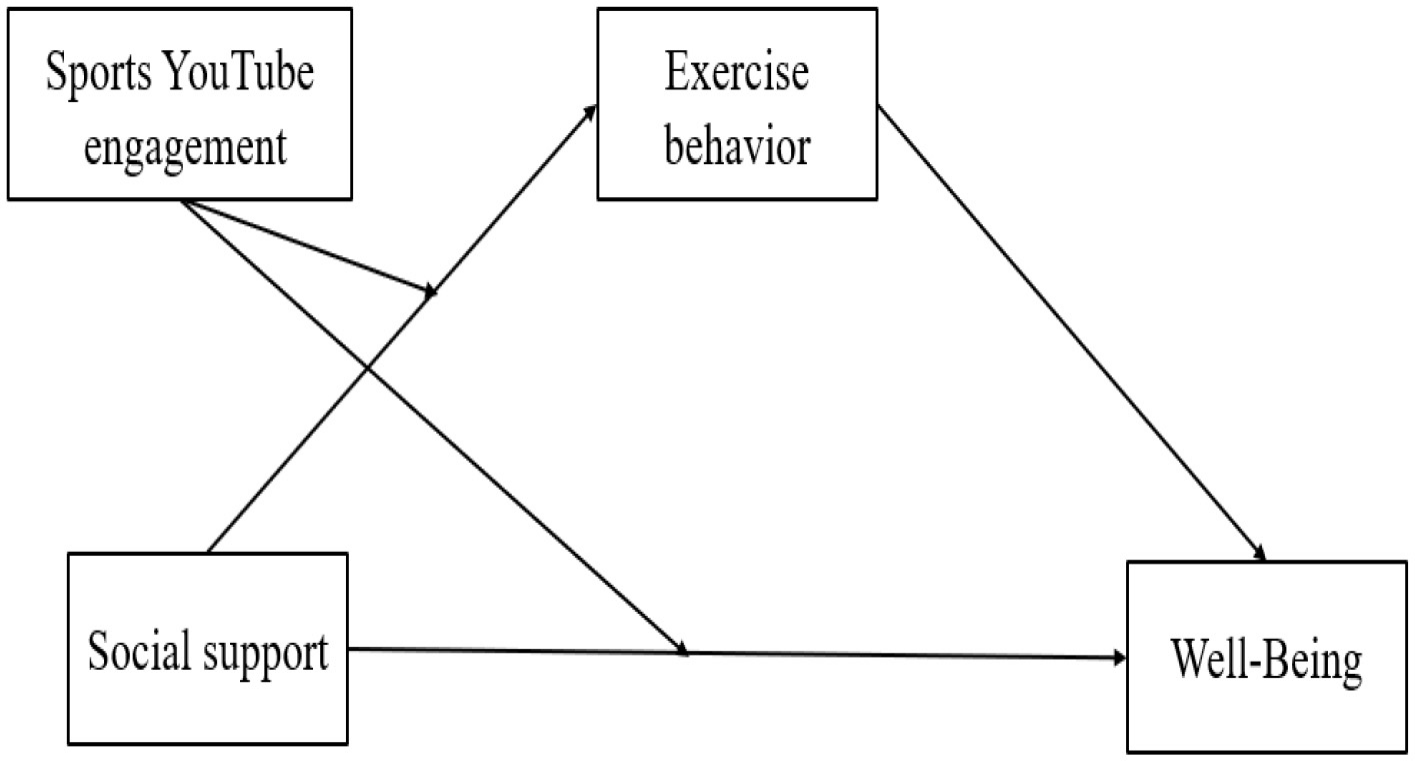
Finalized hypothesized model.

Recent empirical observations further underscore the relevance of imitation in the context of physical activity. The growing accessibility of exercise-related information through digital media platforms has fueled online engagement, as athletes across various disciplines have shared motivational videos featuring home workouts, skill demonstrations, and interactive challenges (Hayes, 2022). These videos, disseminated through platforms such as Instagram, Facebook, and YouTube, serve not only as instructional content but also as imitative models that encourage viewers to replicate physical activity behaviors. Such phenomena reinforce the theoretical underpinnings of Imitation Theory and its applicability to digital health promotion. Moreover, Carpenter et al. (1998) emphasized the role of interest and engagement in imitation as a critical driver of human development. Building upon the frameworks of SCT and Imitation Theory, the present study seeks to investigate the interrelationships among sports YouTube engagement, exercise behavior, and psychological wellbeing. Accordingly, Hypotheses 4 and 5 are proposed to empirically examine these associations. The finalized hypothesized model is presented in Figure 1.

Hypothesis 4: The interaction between social support and sports YouTube engagement will be positively associated with exercise behavior.

Hypothesis 5: The interaction between social support and sports YouTube engagement positively associated with wellbeing.

## 3 Materials and methods

### 3.1 Participants and procedure

This study conducted an online survey in August 2022. The recruitment process targeted students and faculty members at universities located in Jeju Special Self-Governing Province and Gyeonggi Province, South Korea. Participants were recruited through convenience sampling, with recruitment notices and survey links distributed via university online bulletin boards and departmental mobile messaging applications.

To ensure methodological rigor, specific inclusion criteria were applied: participants had to be currently active in at least one university-led sports program and have regular access to sports-related YouTube content. A total of 250 individuals initially accessed the survey link. After excluding 24 responses due to incomplete data or patterned answering (e.g., providing the same response for all Likert-scale items), a final sample of 226 participants was included in the analysis. In accordance with research ethics procedures, only those who voluntarily consented to participate were included in the study. The demographic profile of the participants was described based on the following attributes 160 (70.8%) were male participants, while 66 (29.2%) were female participants. In terms of age distribution, the sample consisted of 50 participants in their 20s (22.1%), 110 in their 30s (48.7%), 48 in their 40s (21.2%), and 18 in their 50s (8%).

### 3.2 Measures

The questionnaire comprised scales with established reliability and validity from previous research. First, social support was measured using 12 items adapted from Miller et al. (1989). In the present study, the scale demonstrated high internal consistency with a Cronbach's alpha of 0.93. Confirmatory factor analysis (CFA) indicated an acceptable model fit (χ^2^ = 28.366, *df* = 9, *p* < 0.001, Q = 3.152, IFI = 0.986, TLI = 0.967, CFI = 0.986, RMSEA = 0.098). Although the RMSEA value approached the.10 threshold, the overall fit was deemed acceptable as the incremental fit indices (CFI, TLI, IFI) significantly exceeded the recommended 0.95 criterion (Hu and Bentler, 1999). According to MacCallum et al. (1996), RMSEA values between 0.08 and 0.10 indicate a mediocre yet permissible fit when other indices demonstrate strong performance. The construct also showed robust composite reliability (CR = 0.91) and convergent validity, with an average variance extracted (AVE) of 0.61.

To measure sports YouTube engagement, a 17-item scale based on Rubin (1981) was utilized. The Cronbach's alpha for this scale was 0.96. The CFA results yielded a satisfactory fit (χ^2^ = 188.282, *df* = 62, *p* < 0.001, Q = 3.037, IFI = 0.962, TLI = 0.943, CFI = 0.961, RMSEA = 0.095), supported by high construct reliability (CR = 0.94) and adequate AVE (0.53). Wellbeing was assessed using eight items from Diener et al. (2010), showing a Cronbach's alpha of 0.90. The CFA indices confirmed an adequate fit (χ^2^ = 32.043, *df* = 12, *p* < 0.001, Q = 2.670, IFI = 0.976, TLI = 0.958, CFI = 0.976, RMSEA = 0.086), which is consistent with contemporary measurement standards (Byrne, 2016). The CR and AVE for wellbeing were 0.94 and 0.68, respectively. All aforementioned items were rated on a 5-point Likert scale, ranging from 1 (strongly disagree) to 5 (strongly agree).

Finally, exercise behavior was assessed using the Godin–Shephard Leisure-Time Exercise Questionnaire (GSLTEQ; Godin and Shephard, 1997). Participants reported their weekly frequency of strenuous, moderate, and light exercise performed for more than 15 mins per session. The total weekly score was calculated as: (9 × strenuous) + (5 × moderate) + (3 × light). This instrument has been validated and consistently applied in studies involving Korean populations (Oh, 2023).

### 3.3 Statistical analysis

The collected data were analyzed using SPSS 24.0, Amos 24.0, and the SPSS PROCESS macro (Model 8; Hayes, 2018) with a significance level of 0.05. Frequency analysis was conducted for demographic characteristics, and Cronbach's alpha was used to assess internal consistency. Construct validity was verified through CFAs, and Pearson's correlation analysis was performed to examine relationships between variables and potential multicollinearity. To test the moderated mediation effect, all continuous variables involved in interaction terms were mean-centered to reduce multicollinearity and improve interpretability. The significance of the conditional indirect effects and the index of moderated mediation were evaluated using 5,000 bootstrap resamples with 95% bias-corrected confidence intervals (CIs). To enhance the internal validity of the model and account for potential confounding effects stemming from the broad age range (20–50 years) of the sample, age was included as a covariate in the analysis.

## 4 Results

### 4.1 Results of statistical and correlation analyses

The descriptive statistics of study variables, including mean, standard deviation, skewness, and kurtosis are listed in Table 1. The data showed normal distribution as skewness and kurtosis fell between recommended ranges, from −2 to + 2 and from −7 to + 7, respectively (Hair et al., 2010). A correlation test was performed to examine overall relationships between variables, and all variables were found to be correlated below 0.70 (see Table 1), confirming an absence of multicollinearity (Bollen and Lennox, 1991).

**Table 1:** Pearson correlations of scores (r), Descriptive statistics.

**Variable**	**1(*r*)**	**2(*r*)**	**3(*r*)**	**4(*r*)**
1. Social support	–	–	–	–
2. Sports YouTube engagement	0.24^**^	–	–	–
3. Exercise behavior	0.15^**^	0.05	-	-
4. Wellbeing	0.59^**^	0.30^**^	0.23^**^	–
Mean	3.77	3.70	55.74	4.06
Standard deviation	0.86	0.96	16.71	0.62
Skewness	−0.34	−0.82	0.98	−0.10
Kurtosis	−0.28	0.24	0.85	−0.99

^**^p < 0.01.

### 4.2 Direct effects of social support, sports YouTube engagement, exercise behavior, and wellbeing

Table 2 presents the direct effects among the variables. The results indicated that social support was positively associated with wellbeing (B = 0.404, *t* = 10.191, *p* < 0.001), thereby supporting Hypothesis 1. In addition, social support was positively associated with exercise behavior (B = 3.554, *t* = 2.657, *p* < 0.01), providing support for Hypothesis 2. Exercise behavior was also positively associated with wellbeing (B = 0.004, *t* = 2.235, *p* < 0.05), confirming Hypothesis 3.

**Table 2:** Direct and moderated mediating effect index.

**Model(s)**		**B**	***SE***	***t***	**95%** ***CI***	
					***LLCI***	***ULCI***
**Model 1: Exercise behavior**						
	Constant	55.167	1.112	49.634^***^	52.977	57.358
Panel A	Social support	3.554	1.338	2.657^**^	0.918	6.191
	Sports YouTube engagement	0.254	1.172	0.216	−2.057	2.564
	Social support ^*^ Sports YouTube engagement	2.872	1.090	2.635^**^	0.724	5.021
**Model 2: Wellbeing**						
	Constant	3.793	0.113	33.656^***^	3.570	4.015
	Social support	0.404	0.039	10.191^***^	0.326	0.482
Panel B	Exercise behavior	0.004	0.002	2.235^*^	0.001	0.008
	Sports YouTube engagement	0.108	0.034	3.172^**^	0.041	0.176
	Social support ^*^ Sports YouTube engagement	0.097	0.032	3.007^**^	0.033	0.161
**Model 3: Conditional indirect effects**						
	Low (-1 SD)	0.004	0.007		−0.010	0.019
Panel C	Medium (Mean)	0.016	0.009		−0.001	0.037
	High (+1 SD)	0.028	0.016		0.001	0.061

Moderator (Sports YouTube engagement) values are categorized as: Low= –0.961 (-1 SD), Medium = 0.000 (Mean), and High = 0.961 (+1 SD). LL, UL: Bias-Corrected 95% Confidence Interval (lower limit, upper limit); ^*^p < 0.05, ^**^p < 0.01, ^***^p < 0.001. Index of moderated mediation = 0.012 (SE = 0.006), 95% CI [0.001, 0.025].

### 4.3 Moderated mediating effects of exercise behavior in the relationship between social support and wellbeing according to sports YouTube engagement

**Figure 2:**
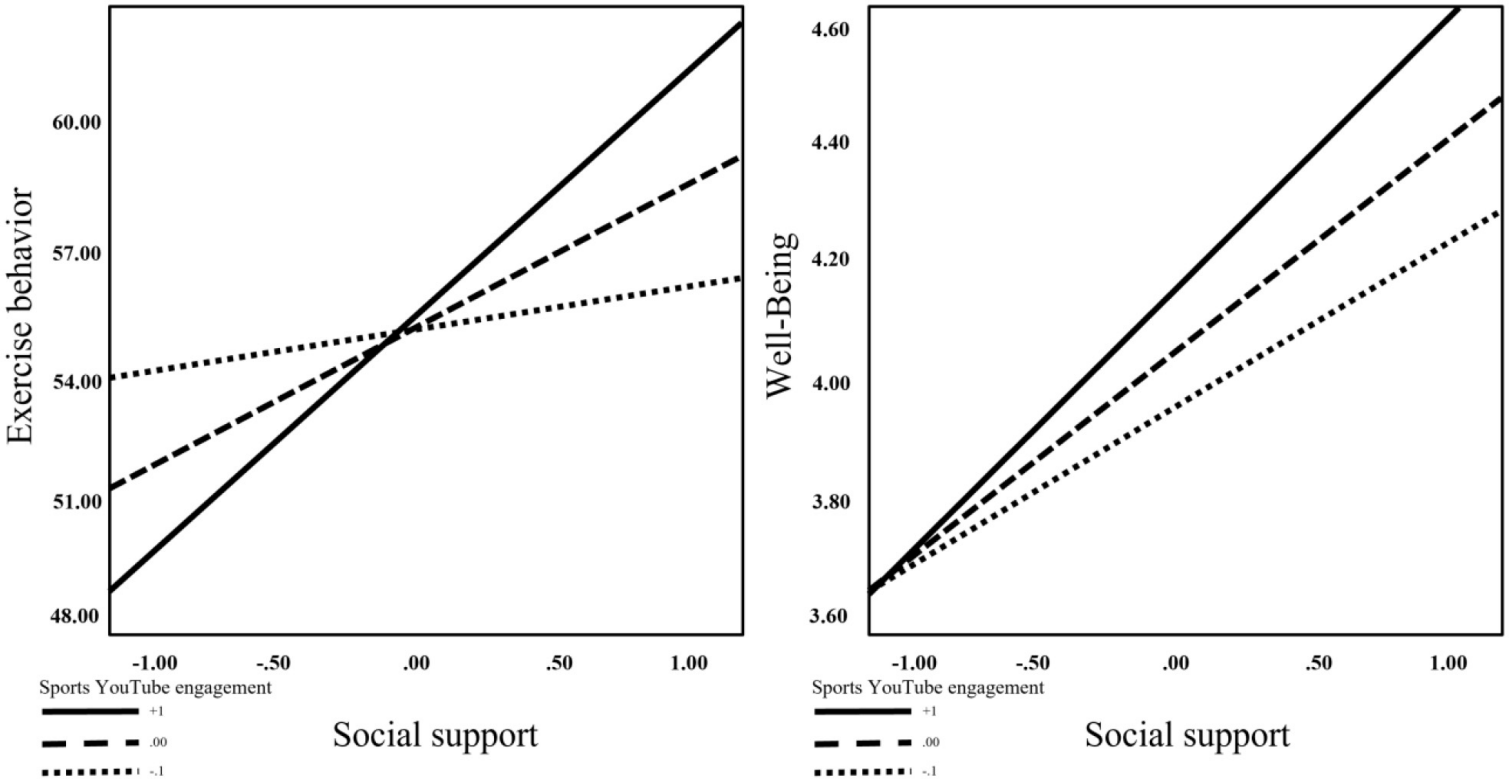
The result of interaction effect.

The interaction between social support and sports YouTube engagement was significantly and positively associated with exercise behavior (B = 2.872, *t* = 2.635, *p* < 0.01). To further clarify this interaction, a simple slope analysis was conducted (as illustrated in Figure 2). The results indicated that the positive relationship between social support and exercise behavior became significantly stronger as the level of sports YouTube engagement increased. Specifically, the interaction effect was significant at both the high level (95% CI = 2.645 to 9.984) and the medium level (95% CI = 0.918 to 6.191), supporting Hypothesis 4.

In addition, the interaction between social support and sports YouTube engagement was positively associated with wellbeing (B = 0.097, *t* = 3.007, *p* < 0.01). As shown in Figure 2, the positive association between social support and wellbeing was stronger at higher levels of YouTube engagement, with significant effects observed at high (95% *CI* = 0.388 to 0.607), medium (95% *CI* =0.326 to 0.482), and low (95% *CI* = 0.223 to 0.398) levels of engagement. These results support Hypothesis 5. The steeper slope at higher engagement levels suggests that sports YouTube engagement acts as a catalyst, amplifying the benefits of social support on both behavioral and psychological outcomes.

Furthermore, the analysis of the indirect effects revealed that the mediating effect of exercise behavior in the relationship between social support and wellbeing was statistically significant only at the high level of sports YouTube engagement (B = 0.028, 95% CI = 0.001 to 0.061). The indirect effects were not significant at the medium (B = 0.016, 95% CI = −0.001 to 0.037) or low (B = 0.004, 95% CI = −0.010 to 0.019) levels, as their confidence intervals included zero. Finally, the index of moderated mediation was formally tested. The result confirmed a significant moderated mediation effect (Index = 0.012, SE = 0.006, 95% CI = 0.001 to 0.025), indicating that the indirect effect of social support on wellbeing through exercise behavior is indeed contingent upon the level of sports YouTube engagement. After controlling for age, the results remained consistent, indicating that the observed relationships between social support, sports YouTube engagement, and exercise behavior are robust across the studied age groups. In addition, to ensure the findings were not driven by outliers, a robustness check was performed by excluding participants with extreme exercise behavior scores. The results showed no significant changes in the overall model, confirming the internal validity of the study.

### 4.4 Gender heterogeneity analysis

To further refine the moderated mediation mechanism a subgroup analysis was conducted by gender. For male participants (*n* = 160), social support was significantly associated with exercise behavior (B = 4.210, *SE* = 1.250, *t* = 3.368, *p* < 0.001) and wellbeing (B = 0.385, *SE* = 0.042, *t* = 9.166, *p* < 0.001). The interaction between social support and sports YouTube engagement also significantly predicted both exercise behavior (B = 3.650, *SE* = 1.120, *t* = 3.258, *p* < 0.01) and wellbeing (B = 0.115, *SE* = 0.038, *t* = 3.026, *p* < 0.01). Most importantly, the index of moderated mediation was statistically significant (Index = 0.016, *SE* = 0.007, 95% CI = 0.004 to 0.032), indicating that for men, sports YouTube engagement effectively amplifies the benefits of social support, facilitating its conversion into actual exercise behavior and psychological wellbeing. In contrast, for female participants (n = 66), a different pattern emerged. While social support remained a robust direct predictor of wellbeing (B = 0.452, *SE* = 0.085, *t* = 5.317, *p* < 0.001), its interaction with sports YouTube engagement did not significantly predict exercise behavior (B = 1.050, *SE* = 1.380, *t* = 0.760, *p* > 0.05) or wellbeing (B = 0.055, *SE* = 0.062, t = 0.887, *p* > 0.05). Consequently, the index of moderated mediation for the female group was not significant (Index = 0.004, *SE* = 0.009, 95% CI = −0.006 to 0.015). These findings suggest that the psychological benefits of social support for female participants are more direct and less contingent upon digital media-driven exercise engagement compared to their male counterparts.

## 5 Discussion

### 5.1 General discussion

The current study investigated the role of sports YouTube engagement associated with individuals' exercise behavior and overall wellbeing. The findings provide compelling evidence that such engagement is associated with enhanced lifestyle and physical activity but also amplifies the positive effects of social support. These results align with and extend the existing literature by confirming all hypothesized relationships among social support, exercise behavior, and wellbeing. Furthermore, the analysis revealed that the mediating role of exercise behavior is contingent upon the levels of sports YouTube engagement and social support. Specifically, individuals with low engagement and limited support exhibited weakened or non-significant associations, suggesting that digital engagement serves as a critical enhancer of health-related outcomes. These insights underscore the growing relevance of online platforms in public health promotion and behavioral change. Grounded in Social Cognitive Theory and Imitation Theory, this research contributes to a deeper understanding of how digital media—particularly sports-focused content—can foster healthier lifestyles.

In this study, we found that social support was significantly and positively associated with wellbeing, confirming Hypothesis 1. This result is consistent with the findings of Siedlecki et al. (2014) and Karademas (2006), who emphasized the importance of interpersonal support in enhancing psychological health. A narrative study by Awang et al. (2014) also demonstrated that social support from family and friends improves wellbeing among university students, which aligns with the present findings. Based on previous research, individuals who receive consistent support from their social networks tend to experience better health outcomes and faster recovery from illness (Hogan et al., 2002; Karademas, 2006). These studies collectively highlight the critical role of social support in fostering psychological resilience and overall wellbeing. By confirming these relationships through empirical analysis, we emphasize the importance of cultivating and maintaining social support. The findings also reinforce the interconnected nature of social support, exercise behavior, and wellbeing, suggesting that these elements work synergistically to promote a healthier and more fulfilling life.

The results of this study indicated that social support demonstrates a significant positive link to exercise behavior, thereby providing empirical support for Hypothesis 2. This finding aligns with prior research conducted by Okun et al. (2003) and Sasidharan et al. (2006), reinforcing the well-established association between social support and physical activity engagement. In line with these studies, we found that social support—defined as the provision of emotional, informational, and instrumental assistance within interpersonal relationships—serves as a key facilitator of exercise behavior. Such support may manifest through encouragement, invitations to participate in physical activity, or logistical help, such as enabling children's involvement in exercise (Gottlieb and Bergen, 2010; Wilson, 2021). Taken together, these findings underscore the pivotal role of social support in both initiating and maintaining regular exercise, suggesting that interventions aiming to enhance physical activity should consider strategies to strengthen supportive social networks.

Next, the findings of this study showed that exercise behavior demonstrates a significant positive link to wellbeing, supporting Hypothesis 3. This result is consistent with previous research demonstrating the psychological benefits of regular physical activity. For instance, Edwards (2006) reported enhanced wellbeing among individuals who engaged in exercise for at least 30 mins, three times per week. Similarly, Edwards et al. (2005) found a significant association between exercise behavior and wellbeing, with physically active individuals exhibiting higher wellbeing scores than those in a non-exercising control group. Furthermore, Zayed et al. (2018) showed that individuals participating in weekly physical activity reported greater life satisfaction and overall wellbeing compared to their sedentary counterparts. However, it is important to note that the effect size observed in the present study was extremely small, indicating limited practical significance. This suggests that, while exercise behavior is reliably related to wellbeing, its direct contribution may be modest within the context of the current model. Taken together, these findings imply that exercise behavior alone may not substantially enhance wellbeing and that broader psychosocial factors such as social support and digital engagement may play a more prominent role in shaping overall wellbeing.

Although a substantial body of research has consistently demonstrated a positive association between exercise and mental health (Salmon, 2001), emerging evidence suggests that this relationship may be influenced by additional contextual factors. While the predominant explanation attributes improvements in wellbeing to the direct psychological and physiological effects of exercise (Mutz et al., 2021; Stubbe et al., 2007), some scholars have cautioned against oversimplifying this link. For example, Brand et al. (2020) emphasized that exercise does not universally result in enhanced wellbeing, highlighting the need to consider moderating variables. In light of this complexity, we sought to examine whether social support and engagement with sports-related YouTube content serve as moderators in the relationship between exercise behavior and wellbeing. By investigating these factors, our study contributes to a more nuanced understanding of how social and digital environments may shape the psychological benefits derived from physical activity.

The findings of this study revealed that the interaction between social support and engagement with sports-related YouTube content showed a significant positive association with both exercise behavior and wellbeing, thereby providing empirical support for Hypotheses 4 and 5. These results align with Lee et al. (2022), who reported that individuals who consumed health-related YouTube videos were 1.33 times more likely to meet recommended physical activity guidelines. Furthermore, our results corroborate those of McDonough et al. (2022), which demonstrated that YouTube utilization positively influenced exercise participation. The broader literature also supports this trend, indicating that engagement with social media platforms—particularly YouTube—is associated with beneficial health outcomes, including increased physical activity and improved dietary adherence (Lee et al., 2022). Importantly, the steeper slope observed at higher levels of engagement suggests that sports YouTube engagement may function as a catalyst, being associated with the amplification of the benefits of social support on both behavioral and psychological outcomes. The current findings should be interpreted within the contextual backdrop of 2022, a period when reliance on digital fitness resources significantly increased (Hayes, 2022). While this study did not directly measure pandemic-related home-exercise habits, the observed strength of sports YouTube engagement suggests that digital platforms have become an integral part of the exercise ecosystem in the post-pandemic era. Building upon these prior studies and our empirical evidence, we suggest that YouTube can serve as a strategic tool for promoting exercise behavior and fostering healthier lifestyles. By integrating social support mechanisms with digital media engagement, individuals may be more likely to initiate and sustain behaviors that contribute to enhanced wellbeing.

In addition to the overall interaction effects, the present study revealed gender heterogeneity in the moderated mediation mechanism linking social support, sports YouTube engagement, exercise behavior, and wellbeing. Specifically, sports YouTube engagement significantly amplified the effects of social support on exercise behavior and wellbeing among male participants, whereas this amplification effect was not observed among females. This pattern is consistent with prior evidence showing that the relationship between social support and leisure-time physical activity varies by gender, suggesting that the conditions under which social support translates into behavioral outcomes may differ across groups (Oliveira et al., 2014). Within this context, social support may increase motivation or readiness to engage in exercise, while sports-related digital content provides accessible and structured guidance that facilitates the translation of such readiness into concrete behavioral action, consistent with Social Cognitive Theory's emphasis on observational learning and guided enactment of behavior (Schwarzer, 2008; Gollwitzer and Sheeran, 2006).

In contrast, the absence of a significant amplification effect among female participants suggests that the benefits of social support may not depend on digitally mediated exercise engagement to the same extent. Rather than indicating weaker effects of social support, this finding implies that the pathways through which social support influences wellbeing may operate differently by gender, with digitally delivered exercise content playing a less central role in shaping these processes for female participants. Taken together, these results underscore that the interaction between social support and digital engagement is conditional rather than universal, highlighting the importance of considering gender as a contextual factor when interpreting moderated mediation mechanisms in digital health and exercise behavior research.

In summary, the findings of the present study underscore the evolving role of social media in promoting physical activity. The interaction between social support and engagement with sports YouTube content showed a significant positive association with both moderate and high levels of physical activity, as well as on psychological wellbeing. In the post COVID-19 era, numerous social media influencers have emerged who use YouTube to provide accessible exercise programs, thereby enabling individuals to participate in physical activity independently. This phenomenon not only reflects meaningful behavioral outcomes but also illustrates the economic potential of digital fitness content creation. From a broader perspective, the continued expansion of sports media, as evidenced by this study, holds promise for advancing interdisciplinary integration between sport psychology and sport management. By leveraging digital platforms and fostering supportive social environments, stakeholders can more effectively design interventions that promote sustainable health behaviors and contribute to the holistic development of the sports industry.

### 5.2 Theoretical and practical implications

This study contributes to both theoretical and practical domains concerning sports YouTube engagement, social support, exercise behavior, and wellbeing. By examining the influence of sports-related YouTube content and social support on physical activity and psychological outcomes, our study offers a more nuanced understanding of the mechanisms underlying these relationships. Specifically, through the integration of Social Cognitive Theory and Imitation Theory, we provide a more robust conceptual framework for interpreting how digital platforms—particularly sports-focused YouTube channels—shape individual health behaviors. These findings expand current knowledge by demonstrating that sports YouTube engagement can serve as an effective tool for health promotion and behavior change, offering valuable insights for researchers, practitioners, and policymakers seeking to leverage social media in public health and sport psychology.

From a practical standpoint, our findings offer meaningful implications for health practitioners and digital health strategists. We argue that integrating sports YouTube engagement into health promotion initiatives can serve as an effective strategy to foster physical activity and enhance overall wellbeing. In particular, collaboration with fitness influencers, certified trainers, and exercise science experts may facilitate the development of compelling and evidence-based content tailored for sports YouTube channels. Such content—ranging from structured workout routines and motivational guidance to scientifically grounded information on the physiological and psychological benefits of exercise—can resonate with diverse audiences. By capitalizing on the broad reach and accessibility of YouTube, health professionals are well-positioned to promote sustainable exercise behaviors and contribute to scalable public health interventions.

In addition, the current study emphasizes the pivotal role of social support in facilitating exercise behavior and enhancing psychological wellbeing. The results of our study suggest that health promotion programs involving physical activity should be designed to actively cultivate social support networks. Strategies such as group-based exercise interventions, peer mentoring systems, and virtual communities might serve as effective platforms where individuals exchange their own experiences, offer mutual encouragement, and share practical advice. By fostering socially supportive environments, these initiatives can help individuals navigate common barriers to physical activity, maintain long-term engagement, and ultimately improve their overall wellbeing. This underscores the importance of incorporating psychosocial components into both digital and community-based health interventions.

The observed gender differences in the moderated mediation mechanism offer important practical implications for the design of digital health and exercise promotion strategies. For male users, the findings suggest that sports YouTube content may be particularly effective when combined with social support mechanisms that emphasize instrumental value, such as skill acquisition, performance improvement, and goal-oriented exercise guidance. Practitioners and content creators targeting male audiences may therefore benefit from integrating instructional workouts, structured training programs, and measurable progress indicators within sports-related YouTube platforms.

In contrast, the absence of a significant amplification effect among female participants implies that digital exercise content alone may be insufficient to enhance wellbeing unless it is embedded within emotionally supportive social contexts. For female users, interventions may be more effective when sports-related digital content is complemented by relational features, such as community-building functions, peer interaction, and emotionally affirming communication. Accordingly, gender-sensitive digital health interventions that differentiate between instrumental and relational pathways may be better positioned to promote sustainable exercise behavior and psychological wellbeing across diverse populations.

### 5.3 Conclusion, limitations and suggestions for future research

In this study, we identified five key findings. First, social support was positively associated with wellbeing. Second, social support showed a significant positive link to exercise behavior. Third, exercise behavior was positively related to wellbeing. Fourth, the interaction between social support and sports YouTube engagement was positively associated with exercise behavior. Fifth, the interaction between social support and sports YouTube engagement demonstrated a positive relationship with wellbeing.

The generalizability of our findings may be constrained by several factors. First, this study focused exclusively on sports-related YouTube engagement and did not examine the potential effects of other social media platforms such as Instagram, TikTok, or Twitter. Because different platforms vary in their content formats and patterns of user interaction, the observed relationships may not extend to other forms of social media engagement. Second, the sample was heavily skewed toward males (approximately 71%), which may limit the applicability of the findings to female participants. Prior research suggests that males and females may differ in their patterns of media engagement (Uhm et al., 2022); therefore, caution is warranted when generalizing the results across genders.

Third, exercise behavior in this study was assessed using a validated composite measure (the GSLTEQ), which captures overall leisure-time physical activity but does not differentiate exercise context or purpose (e.g., home-based vs. facility-based, or skill-focused vs. health-focused activity). As a result, the present findings cannot determine whether the observed mediation effects are driven by specific types of exercise that may be more closely aligned with sports YouTube content. Future research employing multidimensional physical activity measures is needed to disentangle these more nuanced exercise pathways.

Fourth, although prior media engagement research has proposed functional distinctions such as passive consumption, active interaction, and content-specific engagement, these dimensions were not captured by the measurement instrument used in the present study. As a result, the present analysis could not empirically distinguish whether specific forms of engagement differentially contributed to the observed amplification effect. Nevertheless, these theoretical distinctions provide a useful interpretive framework for understanding the findings and offer clear directions for future research employing purpose-built multidimensional engagement scales.

Fifth, this study was conducted within a single cultural context, namely South Korea. Cultural norms and social environments can influence how individuals perceive social support, engage with digital media, and participate in exercise behaviors. Consequently, the mechanisms identified in this study may differ in other cultural settings. Future research should examine these relationships using more gender-balanced samples and across diverse cultural contexts to strengthen the external validity of the findings. Fourth, this study was conducted exclusively with Korean participants, which may limit the cultural generalizability of the results. To address this limitation, subsequent research should include samples from other Asian countries and Western regions to explore potential cultural variations and strengthen the external validity of the findings.

Considering these limitations, we propose that future research explore the differential impacts of various social media platforms on exercise behavior and wellbeing. In particular, examining the unique affordances and user dynamics of platforms beyond YouTube—such as Instagram, TikTok, and Twitter—may yield deeper insights into how digital environments shape health-related outcomes. Additionally, the moderating role of gender in the relationship between social support and health behaviors warrants further investigation, as gender-specific patterns may influence the effectiveness of social interventions. We also recommend that future studies delve into the underlying mechanisms through which social media engagement is associated with exercise behavior and wellbeing. Despite these limitations, our findings provide a foundational understanding of how sports YouTube engagement is linked to physical activity within a specific demographic and cultural framework.

## Data Availability

The raw data supporting the conclusions of this article will be made available by the authors, without undue reservation.
